# Applicability of fibroblast growth factor 23 for evaluation of risk of vertebral fracture and chronic kidney disease-mineral bone disease in elderly chronic kidney disease patients

**DOI:** 10.1186/1471-2369-13-122

**Published:** 2012-09-26

**Authors:** Eiichiro Kanda, Masayuki Yoshida, Sei Sasaki

**Affiliations:** 1 Department of Nephrology, Tokyo Kyosai Hospital, Nakameguro 2-3-8, Meguroku, Tokyo, 153-8934, Japan; 2 Bioethics Research Center, Tokyo Medical and Dental University, Yushima 1-5-45, Bunkyoku, Tokyo, 113-8519, Japan; 3 Department of Nephrology, Tokyo Medical and Dental University, Yushima 1-5-45, Bunkyoku, Tokyo, 113-8519, Japan

**Keywords:** CKD, Fracture, FGF23, CKD-MBD, Phosphate, PTH, Geriatrics

## Abstract

**Background:**

Elderly patients with chronic kidney disease (CKD) are usually at a high risk of fractures due to both osteoporosis and CKD-mineral bone disease (MBD). A new marker is needed to prevent fractures and control CKD-MBD from the early to advanced stages of CKD. In the early stage of CKD, fibroblast growth factor 23 (FGF23) level increases before parathyroid hormone (PTH) and phosphate levels increase, and steadily increases with the progression of kidney disease. It has been reported that FGF23 is related to the overall fracture risk. We investigated the usefulness of FGF23 as a marker for evaluating the risk of vertebral fracture and CKD-MBD in elderly CKD patients.

**Methods:**

One hundred and five elderly predialysis CKD patients who had never been treated for osteoporosis and had never used calcium supplements, vitamin D supplements, or phosphate binders were enrolled in this cross-sectional study in Tokyo, Japan. We investigated the prevalence of vertebral fracture and measured serum calcium, phosphate, 1,25(OH)_2_ vitamin D [1,25(OH)_2_D], intact PTH, FGF23, alkaline phosphatase, and urinary N-terminal telopeptide levels. Then, we examined the relationship between the level of FGF23 and those of bone-metabolism-related markers and identified markers associated with vertebral fractures in elderly CKD patients.

**Results:**

The background features of the patients were as follows: female, 32.4%; diabetes mellitus, 39.0%; average age (standard deviation), 73.2 (7.7) years; and estimated glomerular filtration rate (eGFR), 45.7 (24.1) ml/min/1.73 m^2^. Adjusted multivariate regression analysis showed that the natural logarithm value of FGF23 level [ln(FGF23)] was positively associated with body mass index (*p* = 0.002), serum phosphate level (*p* = 0.0001), and negatively with eGFR (*p* = 0.0006). Multivariate logistic regression analysis showed that vertebral fracture was independently associated with ln(FGF23) (adjusted odds ratio, 4.44; 95% confidence interval, 1.13-17.46). A receiver-operating-characteristic curve of ln(FGF23) showed that the optimal cutoff level of FGF23 indicative of vertebral fracture was 56.8 pg/ml (sensitivity, 0.82; specificity, 0.63).

**Conclusions:**

FGF23 level was independently associated with the levels of bone-metabolism-related markers and vertebral fracture. FGF23 is a new candidate marker for detecting abnormalities of bone metabolism and vertebral fracture in elderly CKD patients.

## Background

Elderly persons are at a high risk of osteoporotic fractures, because of their decreased bone mineral density (BMD). Vertebral fracture is most common [1,2]. There are several studies showing an increased fracture risk among chronic kidney disease (CKD) patients [3-6]. CKD patients tend to show a lower BMD than the general population [7]. Kidney dysfunction is associated with a more rapid decrease in BMD over time [8]. These findings indicate that elderly CKD patients are at a higher risk of fractures because of both CKD and aging than the general population.

CKD affects bone metabolism with a decreased level of the active form of vitamin D, namely, 1,25 dihydroxy vitamin D [1,25(OH)_2_D], and an increased level of the parathyroid hormone (PTH), resulting in CKD-mineral bone disease (MBD) [9]. In CKD, fibroblast growth factor 23 (FGF23) level increases early before PTH and phosphate levels increase, and steadily increases with the progression of kidney disease [10]. The Swedish group of the Population-based Osteoporotic Fractures in Men (MrOS) study, a prospective study, showed that FGF-23 is directly associated with the overall fracture risk and vertebral fracture risk in elderly males with an average estimated glomerular filtration rate (eGFR) of 72.0 ± 20.5 ml/min/1.73 m^2^[11].

Pathological abnormalities of renal osteodystrophy begin in the early stages of CKD [12]. A study on early stage CKD showed that serum calcium and phosphate levels are normal until the eGFR becomes lower than 40 ml/min/1.73 m^2^[13]. Serum phosphate level is not useful for monitoring CKD-MBD at an early stage of CKD, because the changes in serum phosphate level caused by therapy are usually small when the serum phosphate level is within the normal range. We need a new marker of CKD-MBD with higher sensitivity to therapy effects and higher accuracy in identifying patients at a high risk of clinical outcomes of CKD than serum phosphate level at predialysis.

Elderly individuals with CKD often have high risks of both osteoporosis and CKD-MBD. FGF23 level has a potential for identifying early-stage CKD patients that should be targeted for CKD-MBD therapy. For FGF23 level to be clinically useful, it should be demonstrated that FGF23 is a risk factor for the clinical outcomes of CKD, and the target FGF23 level for the prevention of such clinical outcomes should be determined. In this study, we investigated the prevalence of vertebral fracture that occurred within five years in predialysis CKD patients and whether FGF23 level is associated with vertebral fracture.

## Methods

### Study design and study population

This is a cross-sectional study of predialysis CKD patients treated at Tokyo Kyosai Hospital, Tokyo, Japan, which was approved by the Ethics Committee of Tokyo Kyosai Hospital. Patients were eligible for inclusion in the sample for this study when they were at least 60 years of age as of October 1st, 2010, diagnosed as having CKD on the basis of the criteria of the Japanese Society of Nephrology, had never been treated by dialysis or transplantation, had never been treated for osteoporosis, and had never used calcium supplements, vitamin D supplements, phosphate binders, steroids, or immunosuppressants [14]. All the female patients were postmenopausal. Patients who were treated for dementia, congestive heart failure, pulmonary disease, liver disease, or cancer were excluded. We treated CKD in accordance with the CKD practice guideline of the Japanese Society of Nephrology [14]. Body mass index (BMI) was calculated using the following formula: $\text{BMI}=\text{weight}\left(\text{kg}\right)/\text{height}{\left(\text{m}\right)}^{2}$. eGFR was calculated individually using the formula adopted by the Japanese Society of Nephrology using serum creatinine level [14].

### Data

Patient demographics including age, gender, and history of diabetes mellitus, as well as comorbid conditions, were obtained from the medical records of the patients treated at Tokyo Kyosai Hospital. Incident vertebral fractures that occurred within five years, which were diagnosed by orthopedists on the basis of X-ray, computed tomography, or magnetic resonance imaging findings, were also obtained from medical records. Intact PTH and 1,25(OH)_2_D levels were measured as CKD-MBD-related markers. Urinary N-terminal telopeptide (urinary NTX) and alkaline phosphatase (ALP) levels were measured as bone turnover markers [15-17]. Routine serum biochemistry of phosphate, calcium, serum creatinine, urinary creatinine, and intact PTH levels (1–84) was carried out by standard methods at Tokyo Kyosai Hospital. Intact PTH level was measured using an immunoassay system (ARCHITECT PTH, Abbott Japan Co., Chiba, Japan). 1,25(OH)_2_D, urinary NTX, and serum intact FGF23 levels were measured by SRL Inc., Japan. 1,25(OH)_2_D level was analyzed using a commercially available kit, (1,25(OH)_2_D RIA, TFB Co., Tokyo, Japan). Serum intact FGF23 level was measured by enzyme-linked immunosorbent assay (ELISA) with an intra-assay coefficient of variation (CV) of 3.8% at a low standard FGF23 level, an intra-assay CV of 2.4% at a high standard FGF23 level, and an intra-assay CV of 2.8% (Kainos Laboratories International Co., Tokyo, Japan). Urinary NTX level was also analyzed by ELISA (OSTEOMARK, Inverness Medical Co., Chiba, Japan).

### Statistical analyses

Continuous variables for normal distribution were examined by the Kolmogorov-Smirnoff test. Normally distributed variables are presented as mean and standard deviation (SD); otherwise, mean, SD, median and interquartile range (IQR) are presented here. For parameters not normally distributed, natural logarithm values were considered in tests that require normally distributed variables after the tests of their normality: the natural logarithm values of FGF23 level [ln(FGF23)] and PTH level [ln(iPTH)]. Intergroup comparisons were performed using the chi-square test, t-test, and Mann–Whitney U test as appropriate. Pearson’s correlation coefficient, r, was used to assess the relationship between ln(FGF23) and the levels of other bone-metabolism-related markers. Multivariate linear regression analysis was carried out to identify variables that were independently associated with ln(FGF23) by including factors that were previously selected on the basis of Pearson’s correlation coefficient. Univariate logistic regression analysis was performed to identify variables that were associated with vertebral fracture. Then, multivariate logistic regression analysis was performed to identify variables that were independently associated with vertebral fracture by including factors that were previously selected on the basis of the results of the univariate logistic regression analysis. The values of odds ratio (OR) and 95% confidence interval (CI) are summarized. The probability of vertebral fracture occurring in each patient was predicted by calculation using a univariate logistic regression model with ln(FGF23). A receiver-operating-characteristic (ROC) curve was used to determine the optimal cutoff FGF23 level for determining the presence of vertebral fracture, at which optimal sensitivity and specificity were achieved [18]. The cutoff level was determined from the minimal value of (1-sensitivity)^2^ + (1-specificity)^2^ and the Youden index. We defined ln(FGF23) to be high on the basis of the cutoff level determined from the previously obtained ROC curve. The categorized values were treated as binary numbers. We estimated the prevalence of vertebral fracture and calculated the OR of a high ln(FGF23) with respect to normal levels by univariate logistic regression and multivariate logistic regression analyses including factors that were selected on the basis of the results of the previously conducted univariate logistic regression analysis. These analyses were conducted using SAS, version 9.2 (SAS, Inc., Cary, North Carolina). Statistical significance was defined as *p* < 0.05.

## Results

One hundred and five elderly CKD patients were included in the sample for analysis. Patient demographics including biochemical data are shown in Table 1. The causes of CKD were as follows: diabetic nephropathy, 39.0%; nephrosclerosis, 17.3%; chronic glomerulonephritis, 35.6%; and others, 8.1%. Eleven patients were diagnosed as having vertebral fracture (10.5%). Intact PTH and FGF23 levels were log-normally distributed: mean (SD) intact PTH level, 143.6 (139.4) ng/l with a median of 96.2 (IQR, 65.8-154.4); FGF23 level, 78.0 (101.7) pg/ml with a median of 49.0 (IQR, 34.0-71.0).

**Table 1:** Baseline characteristics of patients with vertebral fracture in comparison with those without the fracture

	**All**	**With-fracture group**	**Without-fracture group**	***p*value**
N (%)	105	11 (10.5)	94 (89.5)	
Age (years)	73.2 (7.7)	79.0 (6.6)	72.5 (7.5)	0.008
Female (%)	34 (32.4)	5 (45.5)	29 (30.9)	0.33
Diabetes mellitus (%)	41 (39.0)	4 (36.4)	37 (39.4)	0.84
Height (cm)	159.5 (6.9)	161.1 (3.7)	159.2 (7.2)	0.41
Weight (kg)	61.3 (8.6)	63.2 (2.5)	61.0 (9.2)	0.44
BMI (kg/m^2^)	24.0 (2.5)	24.4 (1.5)	24.0 (2.6)	0.60
eGFR (ml/min/1.73 m^2^)	45.7 (24.1)	32.9 (18.8)	47.2 (24.3)	0.063
Calcium level (mmol/l)	2.4 (0.1)	2.3 (0.2)	2.4 (0.1)	0.85
Phosphate level (mmol/l)	1.1 (0.2)	1.1 (0.2)	1.1 (0.2)	0.81
Intact PTH level (ng/l)	143.6 (139.4) 96.2 (IQR, 65.8-154.4)	142.5 (79.3) 76 (IQR, 45–214)	143.6 (145.1) 91.3 (IQR, 65.1-154.4)	0.29
Ln(iPTH)	4.7 (0.7)	4.8 (0.5)	4.7 (0.7)	0.60
1,25(OH)_2_D level (pmol/l)	122.6 (54.6)	88.3 (49.0)	126.8 (54.0)	0.026
FGF23 level (pg/ml)	78.0 (101.7) 49.0 (IQR, 34.0-71.0)	136.2 (123.2) 120.7 (IQR, 82.2-179.1)	71.2 (97.4) 47 (IQR, 34–66)	0.012
Ln(FGF23)	4.0 (0.7)	4.6 (0.8)	3.9 (0.7)	0.005
ALP level (U/l)	227.0 (83.1)	252.1 (103.5)	224.1 (80.5)	0.29
Urinary NTX level (nmol.BCE/mmol.Cr)	35.8 (24.3)	36.9 (27.3)	35.6 (24.1)	0.88

Values are expressed as mean (SD). The levels of intact PTH and FGF23 are presented with mean (SD), and median (IQR). The levels are compared between the groups by the chi-square test, t-test, or Mann–Whitney U test as appropriate.

Abbreviations: with-fracture group, group with vertebral fracture; without-fracture group, group without vertebral fracture; *BMI* body mass index; *eGFR* estimated glomerular filtration rate; *intact PTH* intact parathyroid hormone; *ln(iPTH)* natural logarithm value of intact parathyroid hormone level; *1,25(OH)*_*2*_*D* 1,25(OH)_2_ vitamin D; *FGF23* fibroblast growth factor 23; *ln(FGF23)* natural logarithm value of fibroblast growth factor 23 level; *ALP* alkaline phosphatase; *urinary NTX* urinary N-terminal telopeptide; *SD* standard deviation; *IQR* interquartile range.

Groups with and without fractures were balanced for gender, diabetes, BMI, eGFR, serum calcium level, serum phosphate level, intact PTH level, ALP level, and urinary NTX level. The average age of the with-fracture group was higher than that of the without-fracture group. The with-fracture group showed a lower 1,25(OH)_2_D level and a higher FGF23 level than the without-fracture group.

### FGF23 and markers of bone metabolism

There was a correlation between ln(FGF23) and the levels of bone-metabolism-related markers (Table 2). Ln(FGF23) was positively associated with age, BMI, serum phosphate level, and ln(iPTH), and urinary NTX level, and negatively with eGFR, serum calcium level, and 1,25(OH)_2_D level. Multivariate regression analysis of the variables age, BMI, eGFR, calcium level, phosphate level, ln(iPTH), 1,25(OH)_2_D level, and urinary NTX level showed that ln(FGF23) was independently associated with BMI, eGFR, and serum phosphate level.

**Table 2:** Ln(FGF23) correlated with levels of bone-metabolism-related markers

	**Pearson’s correlation coefficient**		**Multiple regression analysis**	
	**r**	***p***	**β**	***p***
Age	0.22	0.026	−0.006	0.31
BMI	0.24	0.019	0.056	0.002
eGFR	−0.73	0.0001	−0.012	0.0006
Calcium level	−0.33	0.0006	−0.53	0.23
Phosphate level	0.48	0.0001	0.90	0.0001
Ln(iPTH)	0.68	0.0001	0.16	0.12
1,25(OH)_2_D level	−0.63	0.0001	−0.0022	0.08
ALP level	0.19	0.06		
Urinary NTX level	0.26	0.009	0.0014	0.47

Values are expressed as r (Pearson’s correlation coefficient), β (estimated parameter), and *p* values.

Multiple regression analysis of age, eGFR, calcium level, phosphate level, ln(iPTH), and 1,25(OH)2D level as variables.

Abbreviations: *ln(FGF23)* natural logarithm value of fibroblast growth factor 23 level; *BMI* body mass index; *eGFR* estimated glomerular filtration rate; *ln(iPTH)* natural logarithm value of intact parathyroid hormone level; *1,25(OH)*_*2*_*D* 1,25(OH)_2_ vitamin D; *FGF23* fibroblast growth factor 23; *ALP* alkaline phosphatase; *urinary NTX* urinary N-terminal telopeptide.

### FGF23 and vertebral fracture

**Figure 1:**
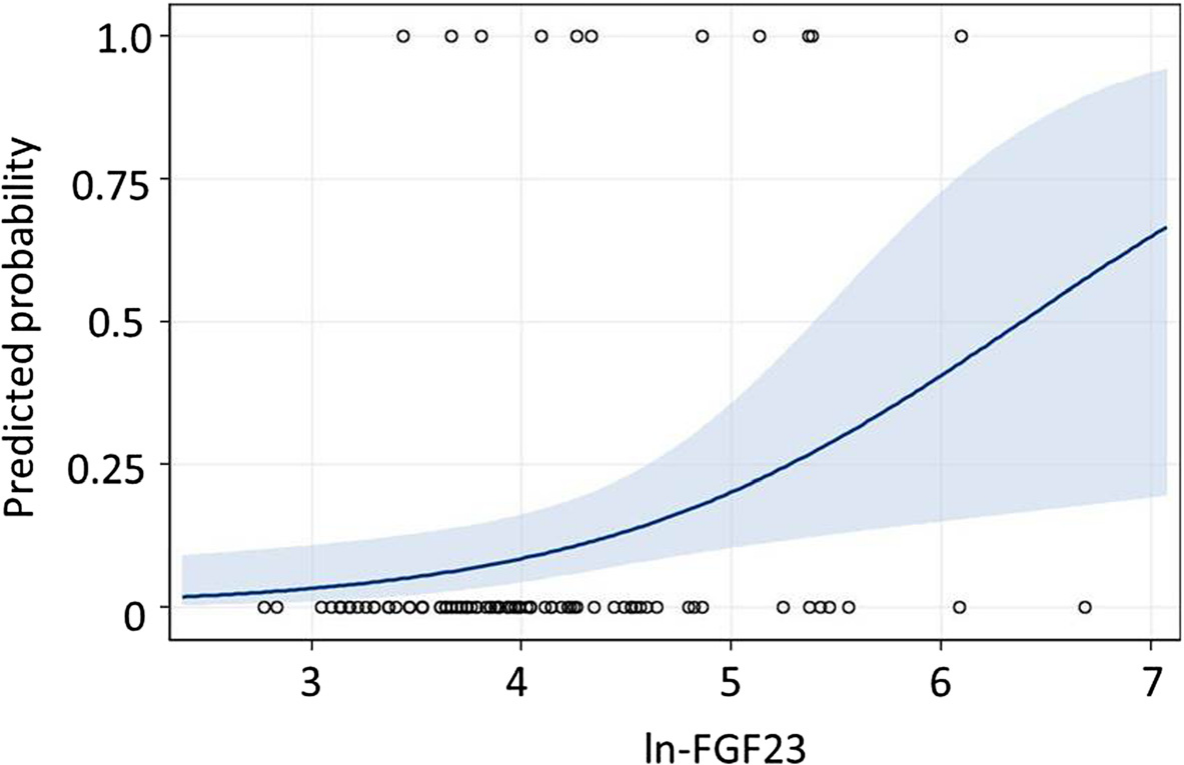
**Predicted probability of detecting vertebral fracture increases with FGF23 level.** A dot shows whether a patient has vertebral fracture (predicted probability = 1) or not (predicted probability = 0). A line shows the predicted probability of detecting vertebral fracture in a patient. The blue area is the 95% confidence interval. Abbreviations: estimated probability, estimated probability of occurrence of vertebral fracture; ln(FGF23), natural logarithm value of fibroblast growth factor 23 level.

The associations between vertebral fracture and the levels of bone-metabolism-related markers are shown in Table 3. Univariate logistic regression analysis showed that vertebral fracture was associated with age, eGFR, and ln(FGF23). The estimated probability obtained using a univariate logistic model with ln(FGF23) was used to assess the effect of FGF23 level on vertebral fracture (Figure 1). The estimated probability of vertebral fracture tended to increase with FGF23 level. A multivariate logistic regression model including age, eGFR, and ln(FGF23) showed that ln(FGF23) was independently associated with vertebral fracture.

**Table 3:** Vertebral fracture correlated with background features, eGFR, and levels of bone-metabolism-related markers

	**Univariate logistic regression OR (95% CI)**	**Multivariate logistic regression OR (95% CI)**
Age (per increase in 1 year)	1.13 (1.03-1.24)	1.13 (1.02-1.26)
Female (Male is reference)	1.87 (0.53-6.62)	
Diabetes mellitus (nondiabetes is reference)	0.88 (0.24-3.22)	
BMI (per increase in 1)	1.07 (0.83-1.37)	
eGFR (per increase in 1 ml/min/1.73 m^2^)	0.97 (0.95-1.00)	1.03 (0.98-1.08)
Calcium level (per increase in 1 mmol/l)	0.63 (0.005-72.0)	
Phosphate level (per increase in 1 mmol/l)	1.38 (0.10-18.86)	
Ln(iPTH) (per increase in 1)	1.38 (0.59-3.23)	
1,25(OH)_2_D level (per increase in 1 pmol/l)	0.98 (0.97-1.00)	
Ln(FGF23) (per increase in 1)	2.70 (1.27-5.74)	4.44 (1.13-17.46)
ALP level (per increase in 1 U/l)	1.00 (0.99-1.01)	
Urinary NTX level (per increase in 1 nmol.BCE/mmol.Cr)	1.00 (0.98-1.03)	

Multivariate logistic regression OR was determined from a logistic regression model that includes age, eGFR, and ln(FGF23) as variables.

Abbreviations: *OR* odds ratio; *CI* confidence interval; *BMI* body mass index; *eGFR* estimated glomerular filtration rate; *ln(iPTH)* natural logarithm value of intact parathyroid hormone level; *1,25(OH)*_*2*_*D* 1,25(OH)_2_ vitamin D; *ln(FGF23)* natural logarithm value of fibroblast growth factor 23 level; *ALP* alkaline phosphatase; *urinary NTX* urinary N-terminal telopeptide.

**Figure 2:**
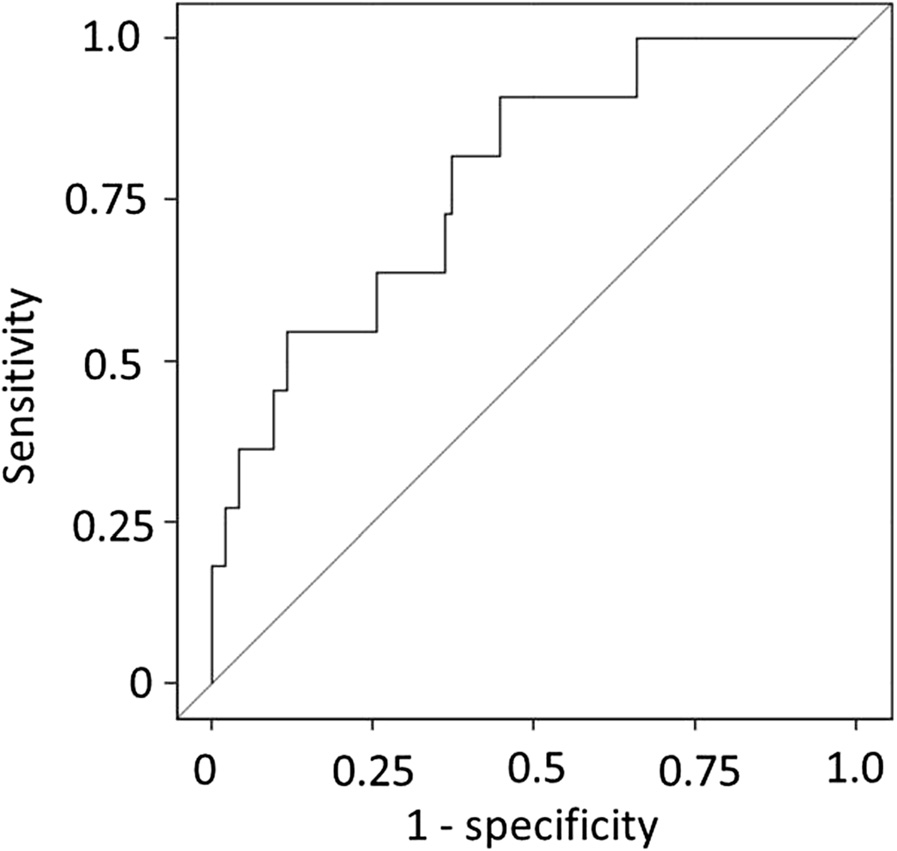
**Receiver operating characteristic curve for ln(FGF23) at optimal cutoff level for predicting vertebral fracture.** Abbreviations: ln(FGF23), natural logarithm value of fibroblast growth factor 23 level.

The ROC curve shows the accuracy of ln(FGF23) in identifying patients at different prevalence rates of vertebral fracture examined in all the participants (Figure 2). At the cutoff level, ln(FGF23) was 4.04 (FGF23 level = 56.8 pg/ml): sensitivity, 0.82; specificity, 0.63; area under the curve (AUC), 0.787. Univariate and multivariate logistic regression analyses showed associations between the likelihood of vertebral fracture and high ln(FGF23): OR, 6.62 (CI, 1.63-26.84); OR adjusted for age and eGFR, 7.91 (CI, 1.09-57.56).

## Discussion

CKD and osteoporosis are common diseases associated with aging, and the number of people with these diseases will increase as will the risk of fractures. However, there are still many unresolved issues about these diseases: the details about bone metabolism in patients with these diseases and the identification of patients with a high risk of fractures. Our findings are consistent with previous reports that the FGF23 level is high in CKD patients with decreased eGFR, increased PTH level, and decreased 1,25(OH)_2_D level [19]. We found that FGF23 level was independently associated with vertebral fracture that occurred within five years in elderly CKD patients. The MrOS study showed prospectively that FGF23 level is related to fracture risk in elderly CKD male patients [11]. In this study, no statistically significant difference in the risk of vertebral fracture was found between genders. These findings can be extended to the association between FGF23 level and vertebral fracture in both sexes with advanced CKD. Moreover, we were able to determine the cutoff FGF23 level for identifying the patients with a high risk of vertebral fracture.

Mutations of FGF23-related genes are associated with several human skeletal disorders. It has been shown that low FGF23 activity due to mutations in *GALNT3*, *FGF23*, and *Klotho* leads to familial hyperphosphatemic tumoral calcinosis, which is characterized by enhanced renal tubular phosphate reabsorption and high serum 1,25(OH)_2_D level in hyperphosphatemia [20-22]. Some types of rickets and osteomalacia such as X-linked hypophosphatemic rickets/osteomalacia, hypophosphatemic rickets/osteomalacia associated with McCune-Albright syndrome/fibrous dysplasia, and tumor-induced rickets/osteomalacia show hypophosphatemia and an increased FGF23 level [23-27]. Overexpressed human FGF23 during osteoblast development in fetal rat calvaria cells suppresses not only osteoblast differentiation but also matrix mineralization independently of its systemic effects on phosphate homeostasis in vitro [28]. A rickets mouse model shows defective osteocyte maturation, an increased FGF23 expression level, and pathological changes in bone mineralization [29]. These clinical and experimental findings suggest that an increased FGF23 level in CKD patients may affect the regulation of bone formation and reabsorption. However, these diseases and manifestations in animal models were also observed in humans or animals with normal kidney function. The role of FGF23 in skeletal mineralization remains unclarified in CKD patients. The high level of FGF23 in CKD is considered to further increase as a compensatory response to maintain phosphate balance because of suppressed renal phosphate excretion or phosphate overload [30,31]. It has been reported that high FGF23 levels are associated with thin osteoids and a short osteoid maturation time in children with renal failure on peritoneal dialysis [32]. Although these findings are not fully applicable to elderly CKD patients, this report suggests that FGF23 indicates the skeletal mineralization status in CKD patients. FGF23 could be a marker of CKD-MBD.

In this study, ROC showed that the cutoff FGF23 level was 56.8 pg/ml. The adjusted logistic regression analysis showed that FGF23 level was an independent factor associated with the prevalence of vertebral fracture that occurred within five years. As far as we researched, there is only one report about the cutoff level for detecting the risk of fractures. The MrOS study showed prospectively that FGF23 levels higher than 55.7 pg/ml are associated with an increased risk of hip and nonvertebral fractures and that FGF23 levels higher than 57.4 pg/ml are associated with an increased risk of vertebral fracture [11]. They used the same ELISA kit as in our study, and these cutoff levels are similar. These two studies differed in the population with regard to age, gender, GFR, and location. Age is an independent risk factor for vertebral fracture [1,2]. GFR is associated with BMD [7,8] and a major predictor of FGF23 level variance in CKD patients [33]. Although these findings indicate the importance of FGF23 level for detecting CKD patients at a high risk of fractures, they do not fully support the validity of these cutoff levels among genreal CKD patients because of the confounding effect of eGFR. The difference in FGF23 level between CKD populations has not been established yet. To determine the cutoff FGF23 level for clinical applications, larger population-based prospective studies need to be carried out.

According to bone biopsy studies, pathological abnormalities in renal osteodystrophy begin in the early stages of CKD [12]. However, the optimal management of CKD-MBD including the monitoring of the levels of markers and therapies initiated at the early stages of CKD has not been established yet. Our study showed that ln(FGF23) was independently positively associated with CKD-MBD-related markers and negatively with eGFR. It has been reported that increased FGF23 levels are associated with an increased risk of progressive CKD independently of normal serum phosphate level [34]. Moreover, an increased FGF23 level is more predictive of CKD progression than serum phosphate level [34]. The changes in serum phosphate level within the normal range tend to not sensitively reflect CKD-MBD in early stages of CKD. On the other hand, FGF23 and PTH levels are often elevated in CKD, but it remains undetermined which increases first and whether the pattern is uniform across all CKD patients. According to the Chronic Renal Insufficiency Cohort Study, FGF23 level increases before PTH and phosphate levels increase in CKD patients [10]. Therefore, FGF23 level can be one of the candidate new markers, because it reflects the progression of CKD-MBD, can be easily followed up longitudinally from the early stages of CKD, and can predict outcomes. The AUC of our study was 0.787, which indicates a moderate accuracy [18]. Although this accuracy may not be very high for FGF23 level to be used as a gold standard for identifying patients with a risk of vertebral fracture, FGF23 level together with the levels of other bone-metabolism-related markers and dual-energy X-ray absorptiometry will be a more useful tool than FGF23 level alone for evaluating CKD-MBD and the risk for fractures and for deciding medications for CKD patients to prevent fractures and progression of CKD-MBD.

This study has several limitations. First, as with any cross-sectional study, we were unable to examine the longitudinal changes in laboratory findings over time. Nonetheless, this study showed that FGF23 level is independently associated with vertebral fractures that already occurred. Second, in this study, we examined 105 patients. The statistical power of this study may not be sufficient for detecting the relationship between fractures and levels of markers. Third, geographical and selection biases may have been included in this study. Fourth, BMD, bone turnover, and mineralization were not measured by an accurate method, such as bone biopsy, in this population. We were unable to evaluate the relationship between FGF23 level and BMD. Fifth, we did not evaluate the medicines used and their effects on the prognosis of kidney function. Sixth, 25-hydroxy vitamin D level was not measured in this study. We were unable to evaluate the CKD patients’ status of vitamin D metabolism. Seventh, only postmenopausal females were enrolled in this study. Thus, we were unable to determine the role of FGF23 in menopausal or premenopausal females.

## Conclusions

For elderly CKD patients, age-related bone diseases and CKD-MBD are significant issues, because such diseases strongly affect their quality of life and mortality. However, there has been no strong evidence that can fully establish indicators that can help guide therapies for bone diseases. We found that the level of FGF23 was associated with those of CKD-MBD-related markers and vertebral fracture in elderly CKD patients. FGF23 level may be a useful marker for detecting and preventing abnormalities of bone metabolism and vertebral fractures from the early to advanced stages of CKD.

## Abbreviations

CKD: Chronic kidney disease; MBD: Mineral bone disease; FGF23: Fibroblast growth factor 23; PTH: Parathyroid hormone; 1,25(OH)_2_D: 1,25(OH)_2_ vitamin D; eGFR: Estimated glomerular filtration rate; ln(FGF23): Natural logarithm value of FGF23 level; BMD: Bone mineral density; MrOS study: The Swedish group of the Population-based Osteoporotic Fractures in Men Study; BMI: Body mass index; Urinary NTX: Urinary N-terminal telopeptide; ALP: Alkaline phosphatase; ELISA: Enzyme-linked immunosorbent assay; CV: Coefficient of variation; SD: Standard deviation; IQR: 25^th^ to 75^th^ intrequartile range; ln(iPTH): Natural logarithm value of intact PTH level; OR: Odds ratio; CI: Confidence interval; ROC: Receiver-operating-characteristic; AUC: Area under the curve.

## Competing interests

No financial or other interests to be declared.

## Authors’ contributions

EK, MY, and SS conceptualized the study and its objective. EK and MY designed the study, extracted the data from the TK database, analyzed the data statistically, and contributed in the interpretation of the results. EK wrote the manuscript. MY and SS revised the manuscript critically and contributed substantially to the content of the article. All the authors read and approved the final manuscript.

## Pre-publication history

The pre-publication history for this paper can be accessed here:

http://www.biomedcentral.com/1471-2369/13/122/prepub
